# Cell polarity as a tumor suppressive mechanism

**DOI:** 10.18632/oncoscience.244

**Published:** 2015-09-14

**Authors:** Maia Al-Masri, Luke McCaffrey

**Affiliations:** Goodman Cancer Research Centre, McGill University, Montreal, QC, Canada

**Keywords:** polarity, breast cancer, proliferation, apoptosis

Cell polarity is a fundamental property of epithelial cells that confers spatial organization to signalling pathways regulating many aspects of cell physiology including survival, proliferation, and motility. Loss of epithelial organization and apical-basal polarity correlates with the acquisition of a malignant phenotype, and accumulating evidence suggests that loss of polarity signalling contributes to cancer progression. [1]

PAR3 is a multi-domain scaffold protein that functions as a central regulator of cell polarity, and is disrupted in multiple cancers. We recently reported that loss of PAR3 promotes tumor growth and metastasis in breast cancer. [2, 3] To promote tumor growth, we observed that loss of PAR3 cooperates with an oncogene to stimulate hyperproliferation by activating Rac1 and its effector Jun N-terminal kinase (JNK). We further found that PAR3 activates RAC1 by deregulating the RAC1-GEF TIAM1, which is normally restricted to tight junctions by PAR3 to spatially regulate RAC1 activity. [2, 4] Interestingly, we found that loss of Par3 alone in normal epithelial cells induced apoptosis, which was dominant over proliferation and limited epithelial growth. [2, 5] However, in the context of an oncogene that suppresses apoptosis, or by pharmacological inhibition of apoptosis, we found that the proliferative effect of PAR3-depletion was uncovered and became dominant. [2] The balance between apoptosis and proliferation is tightly balanced to maintain epithelial homeostasis and this switch in PAR3 function may represent an important switch to promote tumor progression.

This dual effect due to the loss of PAR3 is in some regards similar to a process termed compensatory proliferation, in which proliferation is induced to replace cells eliminated by apoptosis. [6] However, unlike compensatory proliferation, which is induced in response to apoptosis, loss of PAR3 induces apoptosis and proliferation in parallel. [2] This suggests that the order of molecular events in cancer progression is critical. The induction of apoptosis by disruption of polarity in normal tissues therefore acts in a tumor suppressive fashion to eliminate damaged cells. However, disrupting polarity in an environment where apoptosis is suppressed, not only prevents the elimination of cells, but also promotes proliferation of non-polarized cells. This likely contributes to the expansion of non-polarized cells that ultimately populate advanced tumors.

In addition to stimulating proliferation and tumor growth, loss of PAR3 promotes invasion and metastasis. [3, 7] Interestingly, Xue et al. reported that loss of PAR3 activated RAC1 downstream of TIAM1, which loosened cell-cell contacts to promote dissemination of tumor cells. [7] This indicates that the regulation of TIAM1/RAC1 by PAR3 is important to multiple stages of cancer progression. Moreover, we previously reported that PAR3 is required to spatially restrict aPKC in mammary epithelial cells. [3, 5]. Loss of PAR3 in breast tumors also caused mislocalization and activation of atypical PKC, which triggered JAK-dependent activation of STAT3 and induction of matrix metalloproteinase 9 (MMP9) to promote destruction of the extracellular matrix, invasion, and metastasis [3].

**Figure 1:**
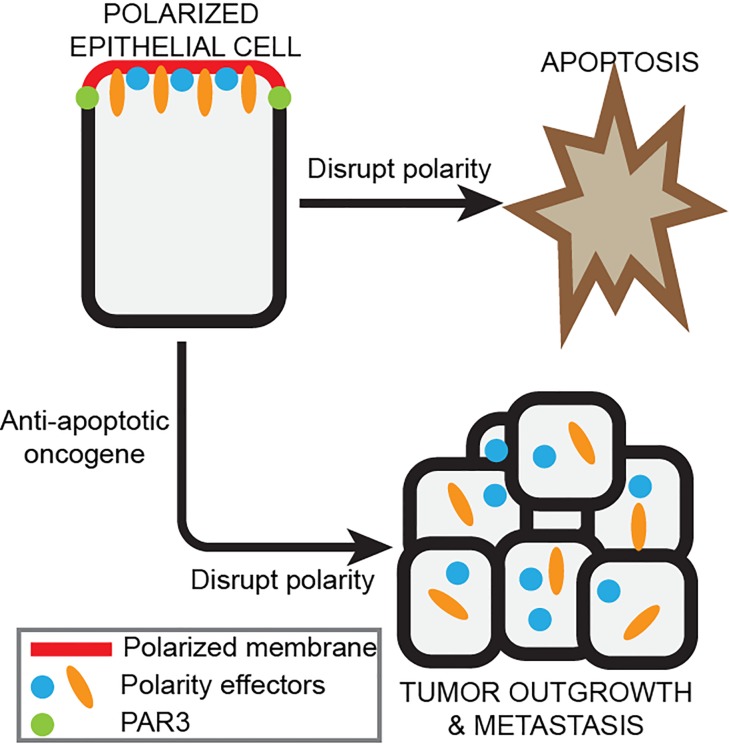
Oncogenic suppression of apoptosis reveals hyperproliferation following disrupted Par3 polarity Loss of PAR3 polarity also promotes invasion and metastasis.

In summary, PAR3 acts as a tumor suppressor for breast cancer. Its function as a polarity scaffold spatially restricts multiple signalling pathways. Loss of this spatial organization deregulates proliferation and invasive programs to promote cancer progression when default apoptotic programs are inhibited. (Figure 1).
